# 5′-(CGA)*_n_* sequence-assisted pH-controlled assembly of supramolecular DNA nanostructure

**DOI:** 10.1098/rsos.180123

**Published:** 2018-08-01

**Authors:** Yuting Yan, Yanwei Cao, Chunsheng Xiao, Yang Li, Xiaoxuan Xiang, Xinhua Guo

**Affiliations:** 1 State Key Laboratory of Supramolecular Structure and Materials, College of Chemistry, Jilin University, Changchun 130012, People's Republic of China; 2 Key Laboratory for Molecular Enzymology and Engineering of the Ministry of Education, College of Life Science, Jilin University, Changchun 130012, People's Republic of China; 3 Key Laboratory of Polymer Ecomaterials, Changchun Institute of Applied Chemistry, Chinese Academy of Sciences, Changchun 130022, People's Republic of China

**Keywords:** CGA DNA sequence, pH-controlled DNA nanostructure, mass spectrometry, DNA assembly

## Abstract

Herein, the DNA strands containing 5′-(CGA)*_n_* and consecutive guanines are used to construct supramolecular DNA nanostructures that are size-controlled by pH values. Additionally, the introduction of thymine linkers within DNA nanostructures is necessary to maintain the stability of long-sized nanostructures. This work also demonstrates a method for accurately building DNA nanostructures.

## 1. Introduction

**Figure 1.**
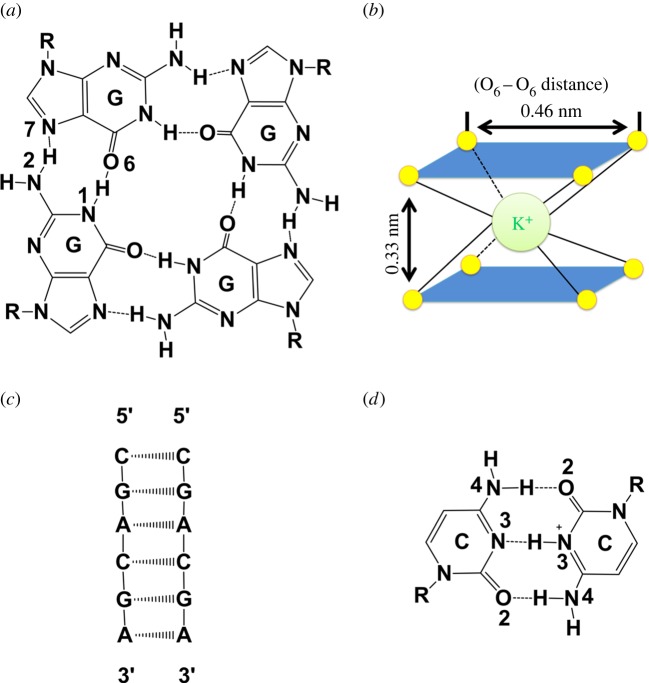
Schematic descriptions of (*a*) a G-quartet linked by Hoogsteen hydrogen bonds; (*b*) the chelation cage formed by eight O_6_ atoms of guanines and K^+^; (*c*) II-DNA formed by d(CGACGA) at pH 4.5; (*d*) C·CH^+^ base pairs formed by cytosine and protonated cytosine.

DNA as one of the important biomolecules demonstrates diversities of conformational polymorphism, including duplexes (B-DNA, A-DNA and Z-DNA), triplexes (C^+^•GC and CG•G) quadruplexes (G-quadruplexes and I-motif), others, etc. [1–5]. G-quadruplexes are tetra-stranded nucleic acid structures formed by G-rich oligonucleotide sequences. Four guanine bases can associate into a G-quartet through Hoogsteen hydrogen bonds, and then two or more G-quartets further assemble into G-quadruplex via hydrophobic stacking (figure 1*a*). The stacking of G-quartets can be stabilized by monovalent or divalent cations like K^+^, NH_4_^+^ and Sr_2_^+^ (figure 1*b*). G-quadruplex has important biological significance in eukaryotic cell telomeres, immunoglobulin switch region and genes promoter region [4]. For duplex, B-DNA is the common conformation formed by Watson–Crick hydrogen bonding [6], while II-DNA is a specific homo base-paired parallel-stranded duplex formed by non-Watson–Crick base pairings [7]. 5′-CGA DNA sequence is a typical motif for self-assembling into II-DNA at a pH lower than 6.0 (figure 1*c*) [7]. At acidic pH, the formation of II-DNA by 5′-CGA sequence is stabilized by C·CH^+^ base pairings (figure 1*d*) and the special interstrand G-A base stack in the GpA step also provides an additional stability force. At neutral or basic pH, the 5′-CGA sequence will lose its ability to form II-DNA structure, but can form conventional B-DNA structure with a complementary sequence.

With remarkable molecular recognition properties, DNA molecules, as promising materials, have been used to build various nanoscale structures in one-, two- and three-dimensional versions, which, in the past 30 years, are receiving growing attention [8,9]. The potential applications of the DNA nanostructures are involved in precise delivery of proteins, drugs or other functional components into designed destination in biological system and use as biosensors for determination of target molecules [8–11]. However, a high cost for DNA sequence synthesis and a high error rate of base mismatch are two prominent problems to be solved for precise construction of DNA nanostructures [10]. G-rich DNA sequences with unique self-assembly capability and feasible formation of rigid and stable G-quadruplexes are considered to be promising for biotechnology and nanotechnology applications [12–20]. The self-assembly of the G-rich sequence, such as d [G_4_T_2_G_4_] and d [G_11_T], could form a long linear structure under certain buffer solution conditions, which was called G-wire or G-lego [15,16]. In addition, the G-quadruplex is also a good building block for constructing various nanostructures. Previously, several DNA sequences were designed and used to form G-quadruplexes connected by duplexes, and the structural motifs could be modulated by changing the types of cations that specifically affect the stabilization of G-quadruplexes [17–22]. Recently, we successfully constructed two supramolecules that contained both G-quadruplexes and i-motifs by enhancing the G-repeat-bearing capacity of i-motifs [21]. Also, a junction DNA nanostructure has been successfully built in lithium acetate buffer solution at a near-neutral pH value via the connection of two slipped junction structures that are formed by G-rich and C-rich strands. The GC-rich duplex junctions in the nanostructure can be switched to G-quadruplexes and i-motifs in weakly acidic potassium acetate solution, which leads to the formation of DNA nanostructures composed of alternating quadruplex and duplex DNA structures [22]. In addition to DNA, there are many other materials that can form self-assembled nanostructures, such as self-assembled dipeptide nanostructures, composites about circularly polarized light and electrospun poly(ϵ-caprolactone)-based fibres [23–25]. However, so far none of these systems use pH-controlled duplexes combined with G-quadruplexes to build length-controllable DNA supramolecular nanostructures.

**Figure 2.**
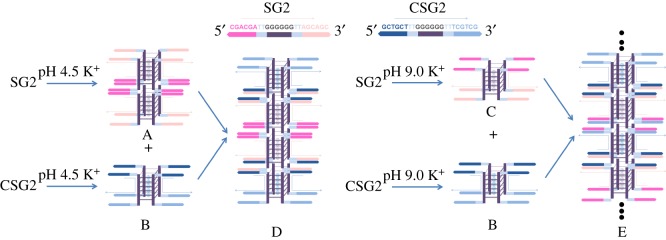
Schematic drawings of postulated DNA supramolecular nanostructures D and E formed by mixing SG2 and CSG2 in KOAc buffer solution at pH 4.5 and 9.0, respectively. The A and C are formed by SG2 sequence d(CGACGATTG_6_TTAGCAGC) in KOAc buffer solution at pH 4.5 and pH 9.0, respectively, while B is formed by CSG2 sequence d(GCTGCTTTG_6_TTTCGTCG) in KOAc buffer solution at both pH 4.5 and pH 9.0.

Previously, we have demonstrated that electrospray ionization mass spectrometry (ESI-MS) is a powerful tool for monitoring stoichiometry of DNA complexes associated by various strands [3,21,22,26] because soft ionization technique of ESI is able to maintain non-covalent interactions of inter-molecules during their transfer from solution to the gas phase. By using ESI-MS, we can obtain accurate information on association preference of G-rich and C-rich DNA strands in various solution conditions [26] and have built a novel DNA nanostructure with both G-quadruplex and i-motif based on ESI-MS determinations [21]. In this work, we attempted to construct G-quadruplex-based supramolecular nanostructures and regulate the assembly of the nanostructures by connecting a specific 5′-(CGA)*_n_* sequence which can form pH-dependent DNA duplex. To this end, four DNA strands were first selected: (i) 5′-CGACGA-3′ (S1), (ii) 5′-TCGTCG-3′(CS1), (iii) 5′-AGCAGC-3′(S2), (iv) 5′-GCTGCT-3′(CS2) (table 1), where S1 is a special sequence forming self-associated II-DNA duplex in acidic condition; S2 has the same base composition as S1 but different base order; S1 and CS1, S2 and CS2 are complementary sequences forming B-type double helixes, respectively. We used ESI-MS, circular dichroism (CD) and UV spectrometry to confirm the conformations formed by the sequences and their mixtures. Following that, we used the sequence d(G_6_) to link S1 and CS1, S2 and CS2, which formed the sequences d(CGACGAG_6_AGCAGC) (SG1) and d(GCTGCTG_6_TCGTCG) (CSG1), respectively; and used d(T_2_G_6_T_2_) to link S1 and S2, CS1 and CS2 which formed the sequences d(CGACGATTG_6_TTAGCAGC) (SG2) and d(GCTGCTTTG_6_TTTCGTCG) (CSG2), respectively (table 1). Thus, the strands SG1, SG2, CSG1 and CSG2 containing consecutive six Gs in the centre were supposed to form stable parallel tetramolecular G-quadruplex core in KOAc buffer solution (structures A–C in figure 2; electronic supplementary material, figure S1*a*–*f*) [26]. Specifically, SG2 could form a G-quadruplex dimer (A in figure 2) connected by II-DNA structure of 5′-(CGA)*_n_* sequences at pH 4.5, and a mixture of SG2 and CSG2 (at a molar ration of 1 : 1) could generate a G-quadruplex tetramer in acidic solution (D in figure 2). In alkaline solution, the mixture would form DNA supramolecular nanostructures with alternating G-quadruplex and B-DNA structure of 5′-(CGA)*_n_* sequences (E in figure 2). These pH-dependent self-assembly properties should be ascribed to the presence of 5′-(CGA)*_n_* that can form II-DNA at acidic pH, but form B-DNA at basic pH. Furthermore, the pH-governed structural transition between G-quadruplex tetramer and the DNA supramolecular nanostructures was also investigated.

**Table 1.** Names and sequences of the single-stranded DNA.

names	sequences	MW
S1	5′-CGACGA-3′	1800.36
CS1	5′-TCGTCG-3′	1782.33
S2	5′-AGCAGC-3′	1800.36
CS2	5′-GCTGCT-3′	1782.33
SG1	5′-CGACGAGGGGGGAGCAGC-3′	5638.99
CSG1	5′-GCTGCTGGGGGGTCGTCG-3′	5602.94
SG2	5′-CGACGATTGGGGGGTTAGCAGC-3′	6856.18
CSG2	5′-GCTGCTTTGGGGGGTTTCGTCG-3′	6820.13

## 2. Experimental section

### 2.1. Material and methods

The DNA sequences (HPLC grade) (table 1) were purchased from Sangon Biotech Co., Ltd (Shanghai, China) and used as received without subsequent purification. Methanol (chromatographic purity), acetic acid (chromatographic purity) and ammonia solution (chromatographic purity) were purchased from Tedia Company, Inc. USA. Potassium acetate, ammonium acetate and other chemical reagents were obtained from Aladdin Biochemical Inc., Shanghai, China. The lyophilized powders of the oligonucleotides were dissolved in Milli-Q water (Millipore, USA) to give stock solutions at a concentration of approximately 1 mM. The precise concentrations of different oligonucleotides were determined based on their UV absorbance at 260 nm at 90°C and the molar absorptivity obtained from the website (http://www.idtdna.com/calc/analyzer). For short DNA sequences (S1, CS1, S2 and CS2), each DNA strand at 100 µM was incubated with pH 4.5 or 9.0 acetate buffer solutions (containing 100 mM ammonium or potassium ions)

Additionally, two pairs of complementary DNA strands (S1 with CS1 and S2 with CS2) were mixed at a molar rate of 1 : 1 in aforementioned acetate buffer solutions. The final DNA concentrations were also maintained at 100 µM. All DNA samples were heated in a 90°C water bath for 10 min and slowly cooled to room temperature, following with the equilibration at 4°C for more than 4 days.

For guanine-contained DNA sequences (SG1, CSG1, SG2 and CSG2), each DNA strand was firstly incubated in potassium acetate solution to ensure the formation of self-assembled G-quadruplexes. After that, the DNA strands with complementary sticking sequences (SG1 with CSG1 and SG2 with CSG2) were mixed in equal molar amounts and equilibrated at 30°C for 3 h. Finally, the mixtures were stored at 4°C to induce further self-assembly for more than 4 days.

### 2.2. Mass spectrometry

Mass spectrometry experiments were carried out by using ESI-Q-TOF (micrOTOF-Q II, Bruker, Bremen, Germany) mass spectrometer at negative ion mode and the data were analysed with Bruker ESI Compass Data Analysis v. 4.0 software. Optimal soft ionization conditions were obtained based on previous methods [21]. This system can measure *m/z* in the range of 50–3000. In order to achieve a satisfactory ionization of the DNA samples, an equal volume of 60% methanol solution was added before MS analysis. The spectrum acquisition time of each sample is 0.6 min and the sample infusing into the ion source is at a rate of 3 µl min^−1^.

### 2.3. Circular dichroism spectroscopy

CD spectra were recorded on a PMS 450 CD spectrometer (BioLogic, France) using 1 mm path length cell at the room temperature. Each sample of 30 µM DNA strand was dissolved in 30 mM KOAc buffer solutions at pH 4.5 and 9.0, respectively. Each spectrum was the average of three scans that recorded from 320 to 200 nm. Each trace was measured at 15 nm min^−1^ of scanning speed with a 2 s acquisition duration at 0.5 nm step. The background spectra corresponding to the buffer alone were subtracted from all DNA spectra.

### 2.4. Ultraviolet absorption spectrophotometry

UV absorbance versus temperature melting curves were measured at 260 nm to acquire the *T*_m_ of various duplexes on a UV-2550 spectrophotometer (SHIMADZU, Kyoto, Japan) equipped with a S-1700 temperature controller [27]. The cell was sealed to avoid solvent evaporation and a magnetic stick was used to dispose of the gas bubbles generated in heating course. Each DNA sample at a concentration of 40 µM was dissolved in 50 mM KOAc at pH 4.5 or 9.0. In each step, the temperature was increased by 1°C and equilibrated for 3 min before recording the absorbance.

### 2.5. Native gel electrophoresis

The native gel electrophoresis was run using 8% polyacrylamide gel. The acidic gel was prepared with 2-(*N*-morpholino) ethanesulfonic acid monohydrate (MES, pH 4.5, 50 mM) while the alkaline gel was prepared by using 1XTBE as the running buffer. The running buffer was further supplemented with 100 mM KCl at pH 4.5 or 9.0. The system temperature was maintained at 4°C by a water circulation system for 3 h at 130 V. The gels were then stained with Gel green DNA staining agent (Biotium) in 0.1 M NaCl, photographed under Ultrapower™ visible light transilluminator (Bioteke, Beijing, China). Each sample contained 100 µM DNA strands and 100 mM potassium ions.

### 2.6. Atomic force microscopy

Atomic force microscopy (AFM) was performed on a Nanoscope IIIa scanning probe microscope (Bruker) from Digital Instruments in the tapping mode with NANOSENSORS™ PPP-NCHR AFM probes. AFM microscopy was performed on the fresh mica surfaces with the help of magnesium ions which can bind negatively charged DNA strands. The DNA samples were annealed at 100 µM in 100 mM K^+^ solution at 4°C for one week. Then aliquots were diluted with 2 mM MgCl_2_ aqueous solution to give a 20 µl analyte containing 1.5 µM DNA. The analytes were spread evenly on the mica surface for 5–8 min. Subsequently, the mica surface was washed with Milli-Q water to wipe off the excess salt, and finally dried in the air.

## 3. Results and discussion

### 3.1. Formation and stability of parallel duplex versus canonical duplex

**Figure 3.**
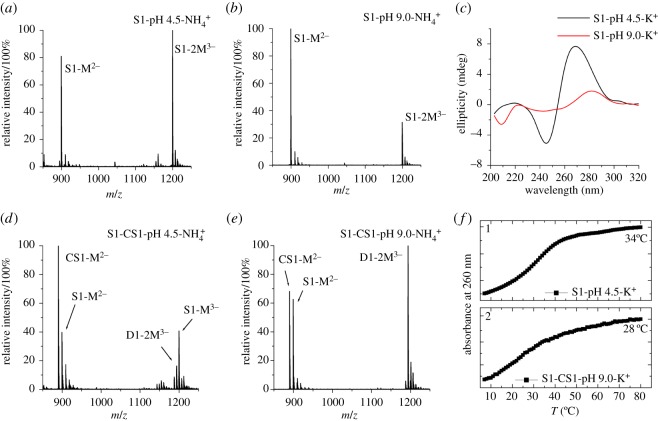
Mass spectra of self-association of S1 in pH 4.5 NH_4_^+^ (*a*) and in pH 9.0 NH_4_^+^ solution (*b*) as well as complementary strand associations of S1 and CS1 in pH 4.5 (*d*) and in pH 9.0 NH_4_^+^ solution (*e*); CD spectra of self-associations of S1 in KOAc buffer solution at pH 4.5 and 9.0 (*c*); the melting processes of S1–CS1 mixtures in KOAc buffer solution at pH 4.5 (*f*1) and pH 9.0 (*f*2). In the mass spectra, S1–M^2−^ represents single-strand S1 ion with two negative charges; S1–2M^2−^ represents double-strand S1 ion with three negative charges; CS1–M^2−^represents single-strand CS1 ion with two negative charges; D1–2M^3−^ represents S1–CS1 complex (B-DNA structure) ion with three negative charges; S1–2M^3−^ represents II-DNA structured S1 ion with three negative charges.

It has been known that 5′-(CGA)*_n_* sequences can self-assemble into parallel duplex structure, called II-DNA, under acidic conditions [7]. Hence, we first studied the formation and stability of II-DNA formed by d(CGACGA) strand (S1 in table 1). As shown in figure 3*a,b*, the abundant bimolecular ions of S1 ([2M]^3−^ at *m/z* 1199.57) were formed in 50 mM NH_4_OAc buffer at pH 4.5, indicating the formation of II-DNA, while most of the bimolecular ions were dissociated at pH 9.0. The formation of II-DNA by S1 in acidic solution was also confirmed by CD spectrum, displaying a sharp positive peak at around 270 nm and an obvious negative peak at around 245 nm (figure 3*c*). However, these CD signatures disappeared when the S1 strands were placed in alkaline solution (figure 3*c*) [28,29]. In comparison, MS and CD spectra of the complementary strand of S1 (CS1) and other DNA strands (including S2 and CS2) indicated the absence of II-DNA in either pH 4 5 or pH 9 buffer (electronic supplementary material, figure S2). These results demonstrated that the II-DNA is specifically formed by 5′-(CGA)*_n_* sequences at acidic pH.

To investigate the structural competition between II-DNA and B-DNA, a mixture of S1 and CS1 with a molar ratio of 1 : 1, was studied by MS and UV spectra. Figure 3*d* shows that [2M]^3−^ ions of S1 with II-DNA structure are more abundant than [2M]^3−^ ions of S1–CS1 complex (D1) with B-DNA structure, indicating that the II-DNA is more stable than corresponding B-DNA under acidic conditions. By contrast, the [2M]^3−^ ions of D1 (at *m/z* 1193.56) became dominant ion species when the mixture was annealed in alkaline solution (figure 3*e*). However, for the reversed sequence S2, the mixture of S2 and CS2 formed B-DNA both in acidic and alkaline conditions (electronic supplementary material, figure S2*d*). UV melting experiments further showed that the *T*_m_ value of II-DNA (34°C) is higher than that of B-DNA (28°C) (figure 3*f*). These results demonstrate that 5′-(CGA)*_n_* are specific sequences that prefer to form II-DNA structure in acidic solution, but form conventional B-DNA structure when they are annealed with complementary strands under basic conditions.

### 3.2. pH-controlled supramolecular assembly of DNA nanostructures

In view of the pH-dependent self-assembly property of S1, we thus connected S1, S2 and their complementary sequences CS1, CS2 with a consequence of consecutive six guanines, in order to investigate the pH-controlled supramolecular assembly of DNA nanostructures. To this end, SG1 (S1–G_6_–S2) and CSG1 (CS2–G_6_–CS1) with completely symmetrical sequences were constructed; meanwhile, SG2 and CSG2 were also constructed by adding TT mismatches in both sides of G_6_ to increase the flexibility of chains (table 1) [21].

**Figure 4.**
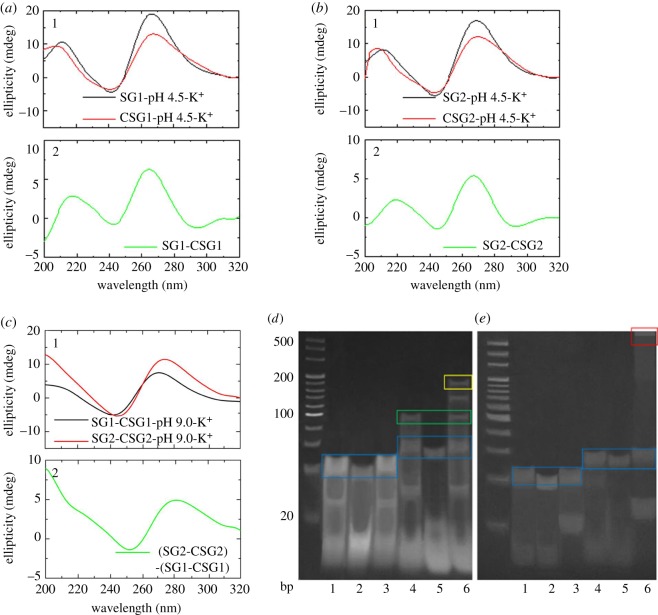
(*a*1) CD spectra for SG1 and CSG1 (30 µM) in a buffer containing 30 mM KOAc at pH 4.5; (*a*2) subtracted CD spectrum obtained by subtracting the CD spectrum of CSG1 from the CD spectrum of SG1. (*b*1) CD spectra for SG2 and CSG2 (30 µM) in a buffer containing 30 mM KOAc at pH 4.5; (*b*2) subtracted CD spectrum obtained by subtracting the CD spectrum of CSG2 from the CD spectrum of SG2. (*c*1) CD spectra for SG2–CSG2 and SG1–CSG1 (30 µM) in a buffer containing 30 mM KOAc at H 9.0; (*c*2) subtracted CD spectrum obtained by subtracting the CD spectrum of SG1–CSG1 from the CD spectrum of SG2–CSG2. (*d*,*e*) Native electrophoretic mobility on 8% polyacrylamide. The DNA strands in buffer solution containing 100 mM KOAc at pH 4.5 (*d*) or pH 9.0 (*e*) were annealed at 4°C for one week: lane 1 (SG1), lane 2 (CSG1), lane 3 (SG1-CSG1), lane 4 (SG2), lane 5 (CSG2), lane 6 (SG2-CSG2). DNA standard markers with 20 bp DNA ladder were purchased from TaKaRa Biotechnology Co., Ltd (Dalian, China).

The self-assembly properties of the designed DNA strands, including SG1, CSG1, SG2 and CSG2, were firstly investigated by CD spectra. Figure 4*a*1,*b*1 and electronic supplementary material, figure S3 show two positive bands at around 215 and 265 nm, and a negative band at around 245 nm in the presence of K^+^, indicating the formation of typical tetramolecular parallel G-quadruplexes [28]. Furthermore, figure 4*a*2 and *b*2 shows the subtracted CD spectra obtained by subtracting the CD spectra of CSG1 and CSG2 from the CD spectra of SG1 and SG2, respectively, which is in accordance with the characteristic CD signatures of II-DNA, indicating the presence of II-DNA under acidic conditions. In this case, the 5′-[CGA]*_n_* residues could play a role of sealing effect preventing further associations of the strands and forming the structure D as shown in figure 2 because of formation of more stable II-DNA under acidic conditions rather than B-DNA under alkaline conditions.

To demonstrate the formation of duplex-connected quadruplex nanostructures under alkaline conditions, the G-quadruplexes of SG1 and CSG1 and G-quadruplexes of SG2 and CSG2 were separately mixed at a molar ratio of 1 : 1. Figure 4*c*1 shows the CD spectra of the mixture of SG1 and CSG1, as well as the mixture of SG2 and CSG2 at pH 9.0. Figure 4*c*2 shows the subtracted CD spectrum obtained by subtracting the CD spectrum of the mixture of SG1 and CSG1 from the CD spectrum of the mixture of SG2 and CSG2, which displays the characteristic CD signatures of B-DNA, including a positive band at around 285 nm and a negative band at around 255 nm. The signature of B-DNA supports the existence of a connection between G-quadruplexes and the formation of supramolecular nanostructures. The addition of TT bases between flanking residues (5′ and 3′) and six consecutive guanines could enhance the flexibility within duplex and G-quadruplex structures during the formation of duplex-connected DNA supramolecular nanostructures [21]. In addition, the CD spectra also indicated a typical II-DNA structure when the mixture of SG2 and CSG2 was incubated at pH 4.5. All the above data suggested that additional bases could provide flexible linkers for the intermolecular interactions of 5′-[CGA]*_n_* sequences, which played a crucial role in the construction of DNA nanostructures.

The native polyacrylamide gel electrophoresis (native-PAGE) experiments were further used to confirm the assumption. Figure 4*d*,*e* shows the migration of various DNA samples under acidic and alkaline conditions, respectively. Lanes 1, 2, 4 and 5 in figure 4*d*,*e* display obvious band at around 40 bp, indicating the formation of tetramolecular G-quadruplexes as shown in electronic supplementary material, figure S1*a*–*e* for SG1, CSG1, SG2 and CSG2 under both pH conditions, which are indicated by blue rectangles. By comparison, lanes 1 and 4 in figure 4*d* show an extra band between 20 and 40 bp, which may be ascribed to the formation of II-DNAs under acidic conditions. In addition, lane 4 in figure 4*d* displays a slower migrating band at around 100 bp, which indicated the formation of larger molecules. We proposed that the structural flexibility caused by TT mismatches may induce the formation of G-quadruplex dimers connected by four pairs of II-DNAs, structure D as shown in figure 2. The bands generated by the mixture of two tetramolecular G-quadruplexes formed by SG2 and CSG2 in higher gel base levels further confirmed our assumption. Lane 6 in figure 4*d* displays three slower migrating bands at around 100, 140 and 180 bp, which should be corresponding to the dimer, trimer and tetramer of G-quadruplexes. This is because G-quadruplex dimers connected by four pairs of II-DNAs can further form four pairs of B-DNAs at each G-quadruplex 3′ ends when the G-quadruplex with complementary residues was mixed together. However, lane 6 in figure 4*e* mainly displays smear bands at the top of the gel when the tetramolecular G-quadruplexes of SG2 and CSG2 were mixed under alkaline conditions, which indicates the formation of ‘G-quadruplex + duplex’-type DNA nanostructures, structure E as illustrated in figure 2. By contrast, no bands corresponding to larger molecules were observed for the mixtures of SG1 and CSG1 in both pH conditions (figure 4*d*,*e*), which indicates that the flexible TT mismatches play a critical role in the formation of DNA supramolecular nanostructures. The bands at the very bottom of the gel (near and below 20 bp) in figure 4*d*,*e* mean the existence of single- and double-stranded DNA formed by short strands that have not been incorporated into supramolecular nanostructures.

**Figure 5.**
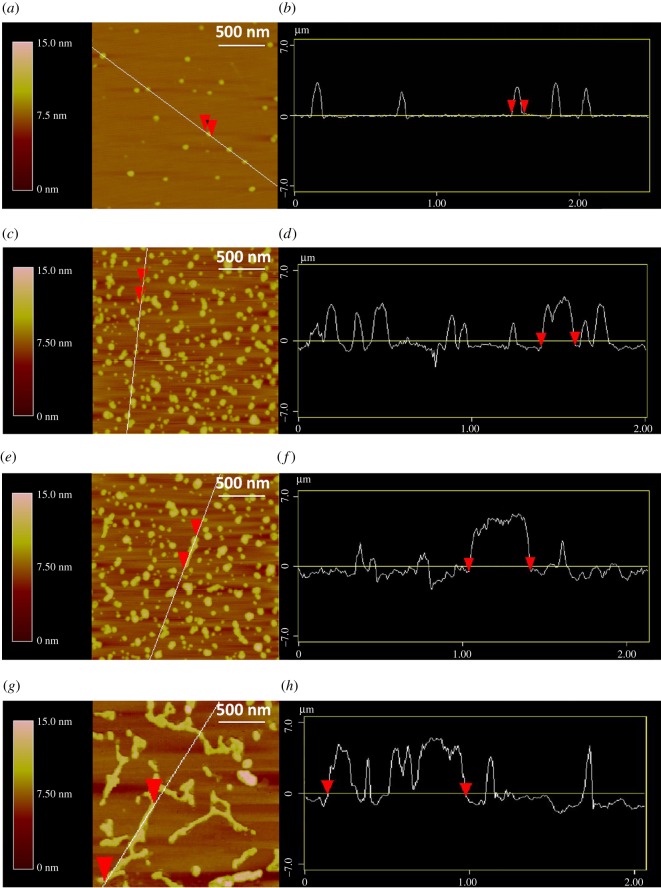
AFM images of the nanostructures formed by DNA G-quadruplexes self-assembly in KOAc buffer solution; (*a*,*b*) SG2 at pH 9.0, (*c*,*d*) SG2 at pH 4.5, (*e*,*f*) a mixture of SG2 and CSG2 at pH 4.5, (*g*,*h*) a mixture of SG2 and CSG2 at pH 9.0. The length of side is 2 µm and the scale bar is 500 nm.

Finally, the formation of DNA supramolecular nanostructures was directly observed through AFM measurements. In figure 5*a,b*, small ellipsoids with sizes of around 3.5 nm in height and 90 nm in length corresponding to single G-quadruplex were observed for the SG2 strand annealed in KOAc buffer solution at pH 9.0 [20]. Similar structures were also observed for the CSG2 samples at both pH 9.0 and 4.5 conditions (electronic supplementary material, figure S5). By contrast, when the SG2 strand was annealed at pH 4.5, some short bar-shaped aggregates with sizes of around 3.8 nm in height and 190 nm in length were observed, which should be corresponding to the dimer of G-quadruplex scaffolds connected by four pairs of II-DNAs. In comparison with aggregates in figure 5*c*, several long bar-shaped aggregates were observed in the mixture of SG2 and CSG2 at pH 4.5 (figure 5*e*). Figure 5*f* demonstrates that these long bar-shaped aggregates are around 4.0 nm in height and 380 nm in length, indicating the formation of tetramer of G-quadruplexes. These results are consistent with the CD experiments. More interestingly, figure 5*g* shows that many long rod-shaped aggregates are visualized in the mixture of SG2 and CSG2 at pH 9.0, which indicates the formation of DNA supramolecular nanostructures. As shown in figure 5*h*, the heights of nanostructures were 4.5 nm on average, while the lengths of them could be up to 1 µm. According to previous reports, these aggregates should be DNA supramolecular nanostructures containing alternating G-quadruplex and duplex structures [19]. Furthermore, the pH-controlled structural transition between G-quadruplex tetramer and the DNA supramolecular nanostructures were also investigated. As shown in electronic supplementary material, figure S6*a*, some long rod-shaped aggregates occurred in the mixture of SG2 and CSG2 when the solution pH was adjusted from 4.5 to 9.0. These results indicated the formation of ‘B-DNA + G-quadruplex’-type supramolecular nanostructures due to the dissociation of II-DNA structures at pH 9.0. By contrast, these rod-shaped aggregates were changed into small ellipsoids and bar-shaped aggregates when the solution pH was modulated to 4.5 (electronic supplementary material, figure S6*b*). These results demonstrate that the assembly and dissociation of DNA nanostructures can be easily controlled by pH, which is promising for constructing ‘DNA machines’ [10].

## 4. Conclusion

We have demonstrated that the connection of duplex-forming sequences with a G-quadruplex-forming sequence (G6) could be used to construct DNA supramolecular nanostructures with alternating B-duplex and G-quadruplex structures. Our results demonstrate that the TT linker between B-duplex and G-quadruplex structures are necessary for the construction of such nanostructures, because the TT linker can provide structural flexibility for the bending of duplexes at the terminal of G-quadruplex. However, interestingly, the size of self-assembled nanostructures could be modulated by solution pH, because the 5′-(CGA)*_n_* sequences could form stable parallel II-DNA under acidic conditions, but form B-DNA in basic solution. In general, it is the first time to take advantage of the pH-dependent self-assembly feature of 5′-(CGA)*_n_* sequences for building supramolecular DNA nanostructures, and this pH-dependent self-assembly of DNA may be promising for constructing DNA machines. Also, the method demonstrated here could be widely used for constructing accurate DNA nanostructures.

## Supplementary Material

Electronic Supplementary Information for 5'-(CGA)n sequence-assisted pH controlled assembly of supramolecular DNA nanostructure

## Supplementary Material

Electronic Supplementary Information for 5'-(CGA)n sequence-assisted pH controlled assembly of supramolecular DNA nanostructure

## Supplementary Material

Electronic Supplementary Information for 5'-(CGA)n sequence-assisted pH controlled assembly of supramolecular DNA nanostructure

## Data accessibility

The datasets supporting this article have been uploaded as part of the electronic supplementary material and also deposited at Dryad Digital Repository (http://dx.doi.org/10.5061/dryad.55t3fb5) [30].
