# Supplementary material for: 5′-(CGA)n sequence-assisted pH-controlled assembly of supramolecular DNA nanostructure
Source: R Soc Open Sci. 2018 Aug 1;5(8):180123. doi: 10.1098/rsos.180123 (PMC6124056; doi:10.1098/rsos.180123)
Supplement: Electronic Supplementary Information for 5'-(CGA)n sequence-assisted pH controlled assembly of supramolecular DNA nanostructure [file rsos180123supp1.doc]

Electronic Supplementary Information for

**5'-(CGA)_n_ sequence-assisted pH controlled assembly of supramolecular DNA nanostructure**

Yuting Yan^a^, Yanwei Cao^a^, Chunsheng Xiao^*,b^, Yang Li^a^, Xiaoxuan Xiang^a^, Xinhua Guo^*,a, c^

^a^ State Key Laboratory of Supramolecular Structure and Materials, College of Chemistry, Jilin University, Changchun 130012, China

^b^ Key Laboratory of Polymer Ecomaterials, Changchun Institute of Applied Chemistry, Chinese Academy of Sciences, Changchun 130022, China

^c^ Key Laboratory for Molecular Enzymology and Engineering of the Ministry of Education, College of Life Science, Jilin University, Changchun130012, China

Corresponding Authors

Dr. Xinhua Guo, E-mail: [guoxh@jlu.edu.cn](mailto:guoxh@jlu.edu.cn) ;

Dr. Chunsheng Xiao, Email: [xiaocs@ciac.ac.cn](mailto:xiaocs@ciac.ac.cn)


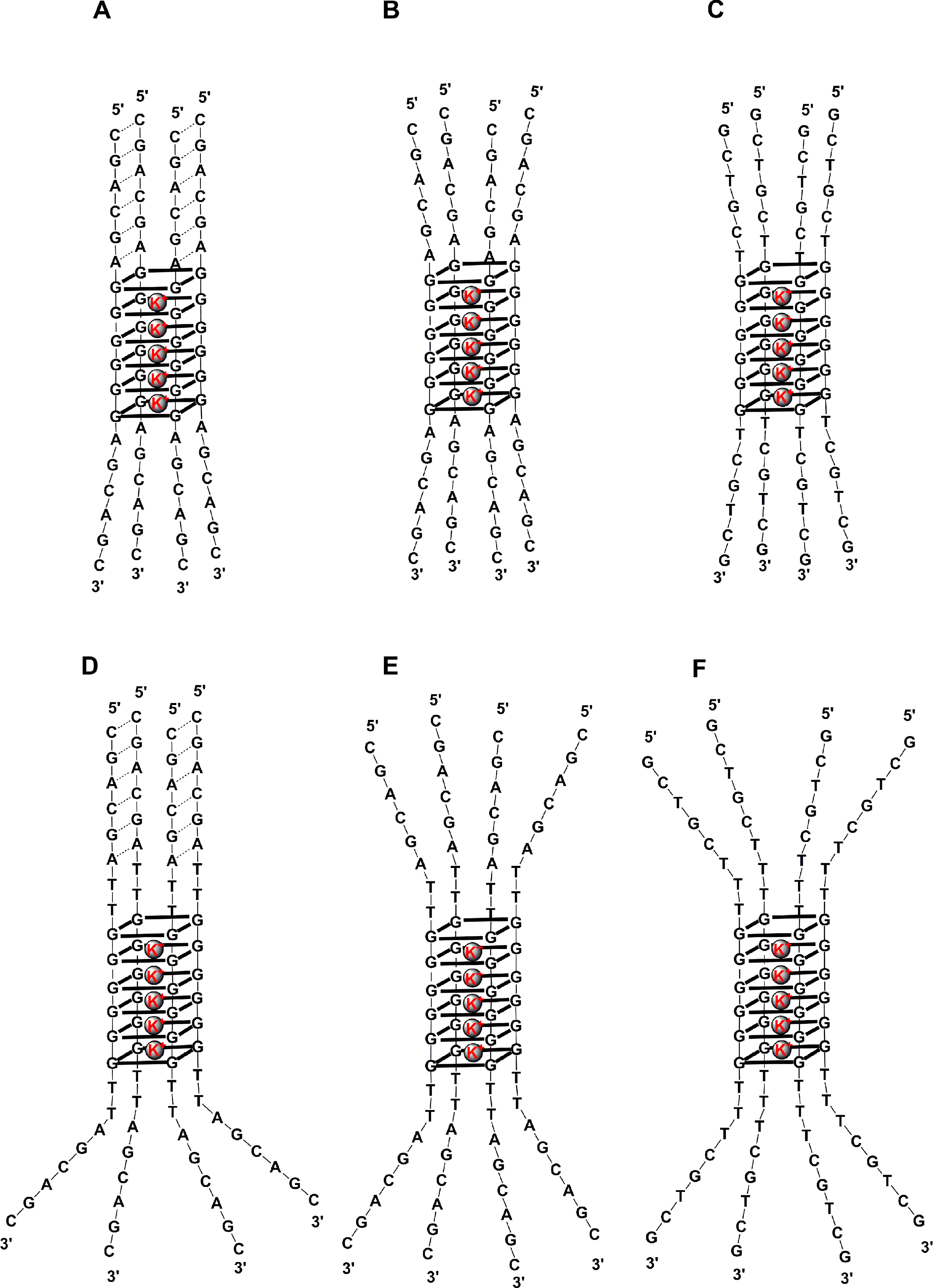


Fig. S1. Schematic drawings of postulated G-quadruplex monomer structures formed by (A) SG1 at pH 4.5, (B) SG1 at pH 9.0, (C) CSG1 at pH 4.5 or 9.0, (D) SG2 at pH 4.5, (E) SG2 at pH 9.0, (F) CSG2 at pH 4.5 or 9.0.


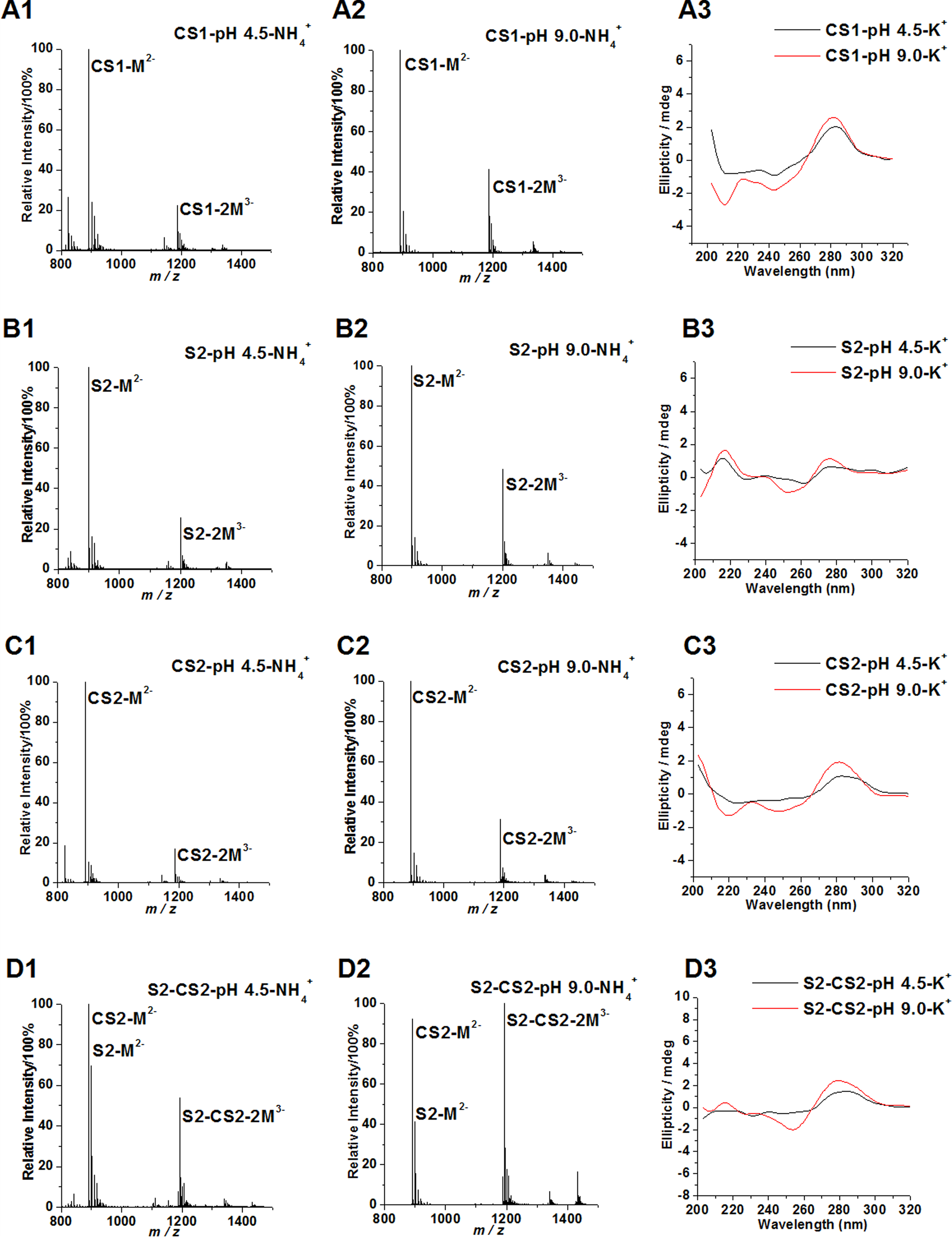


Fig. S2. Mass spectra of oligonucleotides CS1 (A1 and A2), S2 (B1 and B2), CS2 (C1 and C2) and S2-CS2 (D1 and D2) in 50 mM NH_4_OAc buffer solution at pH 4.5 and 9.0 respectively; CD spectra of oligonucleotides CS1 (A3), S2 (B3), CS2 (C3) and S2-CS2 (D3) in 30 mM KOAc buffer solution at pH 4.5 and 9.0; after incubating the samples at 4°C for 4 days.


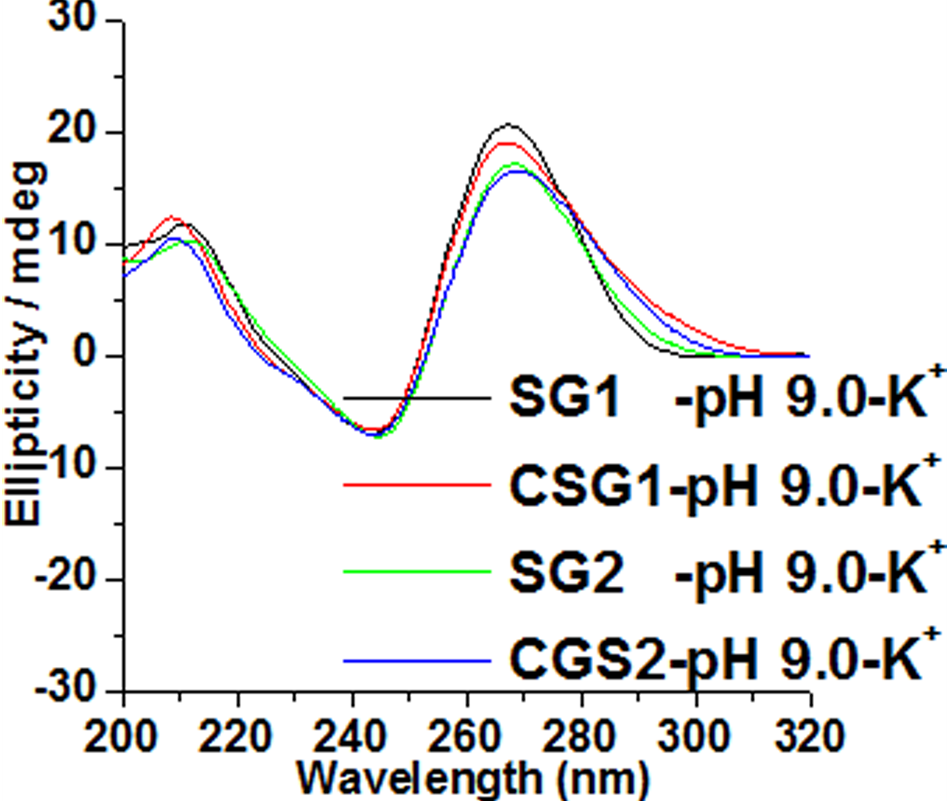


Fig. S3. CD spectra of the oligonucleotides SG1, CSG1, SG2 and CSG2 in 30 mM KOAc buffer solution at pH and 9.0.


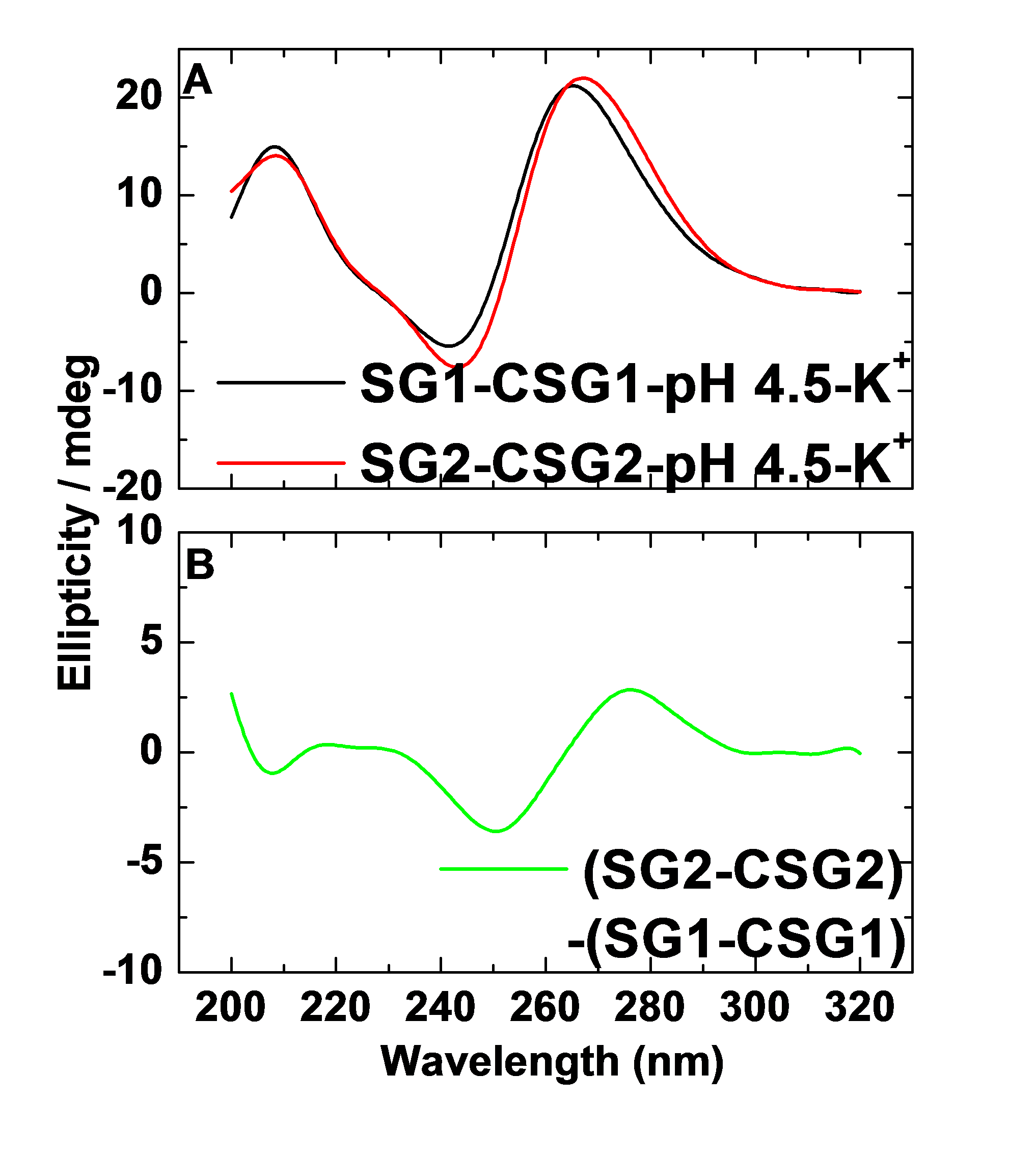


Fig. S4. (A) CD spectra for SG2-CSG2 and SG1-CSG1 (30 μM) in a buffer containing 30 mM KOAc at pH 4.5. (B) Subtracted CD spectrum obtained by subtracting the CD spectrum of SG1-CSG1 from the CD spectrum of SG2-CSG2 in Figure S4A.


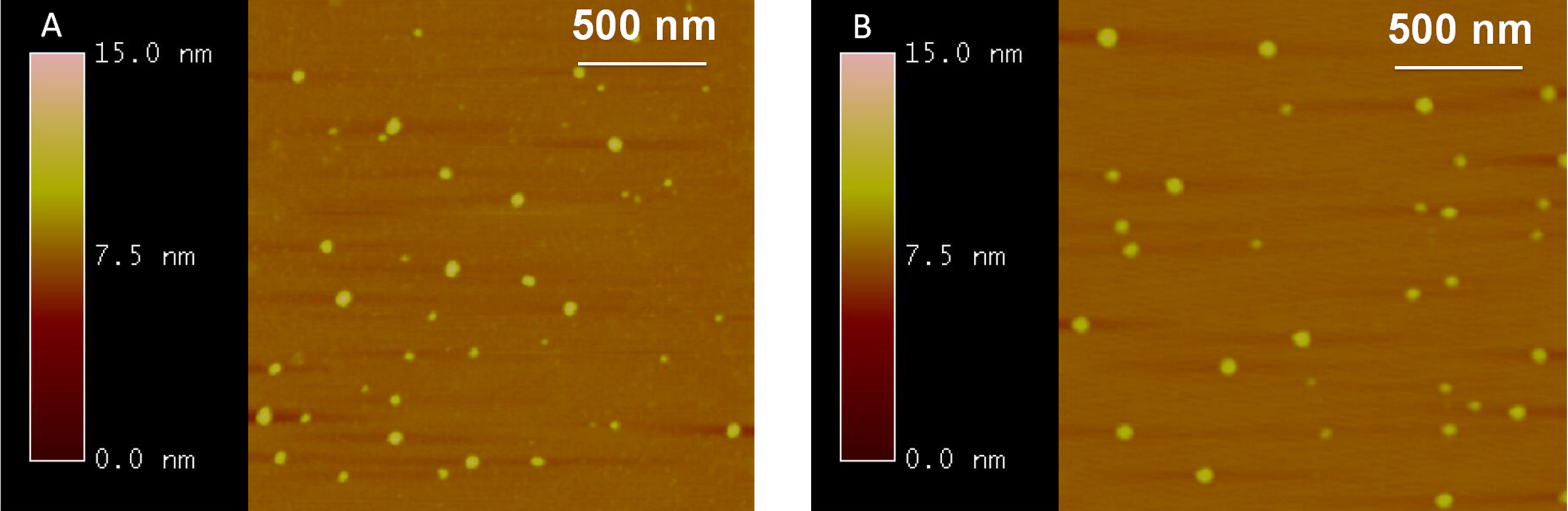


Fig. S5. AFM images of DNA G-quadruplexes self-assembly by oligonucleotide sequences (A) CSG2 at pH 4.5, (B) CSG2 at pH 9.0. The samples in KOAc buffer were deposited on the freshly cleaved mica substrate and subjected to AFM measurements. The scale bar is 500 nm. The square shows the relative height of spots.


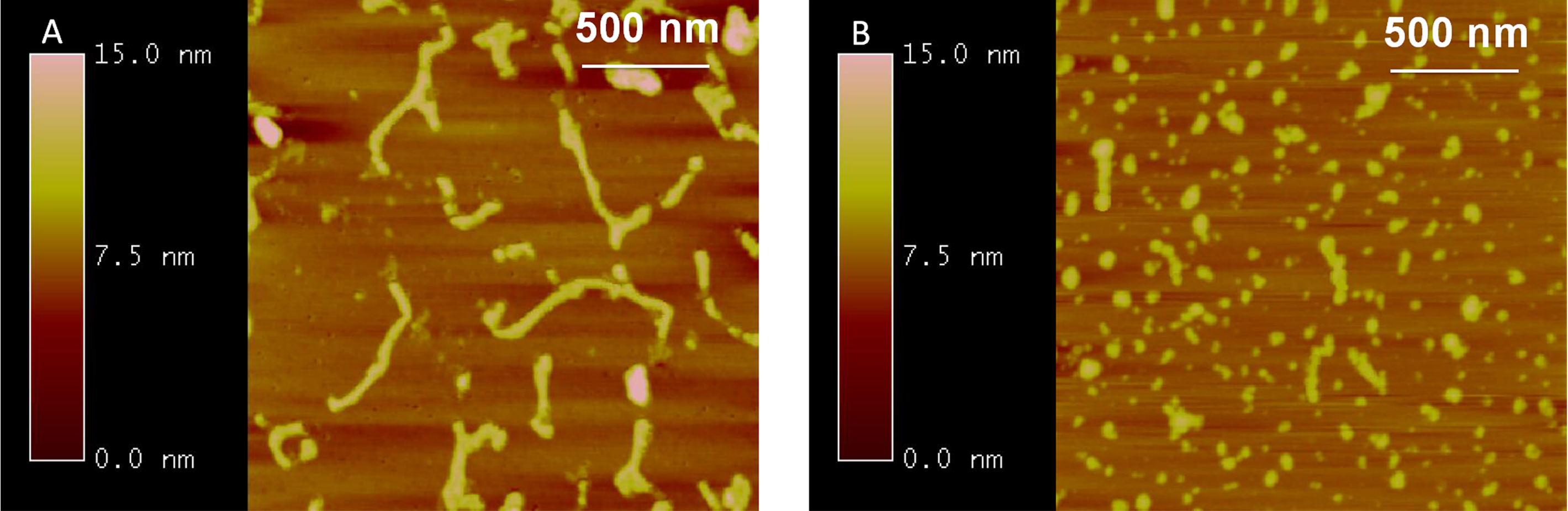


Fig. S6. AFM images of the mixture of SG2 and CSG2 when the solution pH was adjusted from 4.5 to 9.0 (A), and when the solution pH was modulated from 9.0 to 4.5 (B). The samples in KOAc buffer were deposited on the freshly cleaved mica substrate and subjected to AFM measurements. The scale bar is 500 nm. The square shows the relative height of spots.
